# Evolution of blood magnesium and phosphorus ion levels following thyroidectomy and correlation with total calcium values

**DOI:** 10.1590/S1516-31802010000500005

**Published:** 2010-09-02

**Authors:** Alexandre de Andrade Sousa, José Maria Porcaro Salles, João Marcos Arantes Soares, Gustavo Meyer de Moraes, Jomar Rezende Carvalho, Paulo Roberto Savassi-Rocha

**Affiliations:** I MD. Surgeon in the Head and Neck Group and member of the Instituto Alfa de Gastroenterologia (IAG), Hospital das Clínicas, Universidade Federal de Minas Gerais (UFMG), Belo Horizonte, Minas Gerais, Brazil.; II MD. Professor of Head and Neck Surgery and Head of the Head and Neck Group of the Instituto Alfa de Gastroenterologia (IAG), Hospital das Clínicas, Universidade Federal de Minas Gerais (UFMG), Belo Horizonte, Minas Gerais, Brazil.; III MD, PhD. Surgeon in the Head and Neck Group and member of the Instituto Alfa de Gastroenterologia (IAG), Hospital das Clínicas, Universidade Federal de Minas Gerais (UFMG), Belo Horizonte, Minas Gerais, Brazil.; IV MD, PhD. Professor of Surgery and Head of the Instituto Alfa de Gastroenterologia (IAG), Hospital das Clínicas, Universidade Federal de Minas Gerais (UFMG), Belo Horizonte, Minas Gerais, Brazil.

**Keywords:** Magnesium, Phosphorus, Calcium, Thyroidectomy, Complications, Magnésio, Fósforo, Cálcio, Tireoidectomia, Complicações

## Abstract

**CONTEXT AND OBJECTIVE:**

Magnesium ion concentration is directly related and phosphorus ion concentration is inversely related to calcemia. The aim of this study was to evaluate the evolution of magnesium and phosphorus ion levels in patients undergoing thyroidectomy and correlate these with changes to calcium concentration.

**DESIGN AND SETTING:**

Prospective study at the Alpha Institute of Gastroenterology, Hospital das Clínicas, Universidade Federal de Minas Gerais.

**METHODS:**

The study included 333 patients, of both genders and mean age 45 ± 15 years, who underwent thyroidectomy between 2000 and 2005. Total calcium, phosphorus and magnesium were measured in the blood preoperatively and 24 and 48 hours postoperatively. Ionic changes were evaluated according to the presence or absence of postoperative hypocalcemia.

**RESULTS:**

There were statistically significant drops in blood phosphorus levels 24 and 48 hours after thyroidectomy, compared with preoperative values, in the patients without hypocalcemia. In the patients who developed hypocalcemia, there was a significant drop in plasma phosphorus on the first postoperative day and an increase (also statistically significant) on the second day, in relation to preoperative phosphorus levels. A significant drop in postoperative magnesium was also observed on the first and second days after thyroidectomy in the patients with hypocalcemia, in relation to preoperative levels. In the patients without hypocalcemia, the drop in magnesium was significant on the first day, but there was no difference on the second day.

**CONCLUSION:**

Despite the postoperative changes, neither magnesium nor phosphorus ion levels had any role in post-thyroidectomy calcemia.

## RESUMO

**CONTEXTO E OBJETIVO:**

A concentração do íon magnésio está diretamente e a do íon fósforo inversamente relacionada à calcemia. O objetivo foi avaliar a evolução das concentrações dos íons magnésio e fósforo nos pacientes submetidos a tireoidectomia, e relacioná-los com as alterações da concentração do cálcio.

**TIPO DE ESTUDO E LOCAL:**

Estudo prospectivo realizado no Instituto Alfa de Gastroenterologia, Hospital das Clínicas, Universidade Federal de Minas Gerais.

**MÉTODOS:**

O estudo incluiu 333 pacientes de ambos os sexos, com média de idade de 45 ± 15 anos, submetidos a tireoidectomia no período de 2000 a 2005. Cálcio total, fósforo e magnésio foram dosados no pré-operatório e com 24 e 48 horas de pós-operatório. As alterações dos íons foram avaliadas de acordo com a presença ou ausência de hipocalcemia pós-operatória.

**RESULTADOS:**

Houve queda estatisticamente significativa dos níveis sanguíneos de fósforo 24 horas e 48 horas após a tireoidectomia em relação ao pré-operatório nos pacientes que não tiveram hipocalcemia. Quanto aos pacientes que evoluíram com hipocalcemia, houve queda significativa do fósforo plasmático no primeiro dia de pós-operatório e elevação, também estatisticamente significativa, no segundo dia em relação ao fósforo pré-operatório. Foi também observada queda significativa do magnésio pós-operatório em relação ao pré-operatório no primeiro e no segundo dia após a tireoidectomia nos pacientes com hipocalcemia. Naqueles sem hipocalcemia, a queda do magnésio foi significativa no primeiro dia, mas não houve diferença no segundo dia.

**CONCLUSÃO:**

Embora alterados no pós-operatório, as concentrações dos íons magnésio e fósforo não apresentaram papel na calcemia pós-tireoidectomia.

## INTRODUCTION

Thyroidectomy is a clean surgical procedure with a small exposed area, minimal blood and fluid loss and no involvement of the digestive tract. Dietary intake can be resumed a few hours after the operation, in most cases. The endocrine and metabolic response is slight and short-lived. Consequently, both fluid volumes and ion concentrations promptly return to their baseline preoperative states.^1-3^

Although the incidence of postoperative complications is acceptable, they may be extremely uncomfortable and incapacitating. The most common metabolic complications are disorders of calcium ion concentrations.^4^

Homeostasis of magnesium ions is directly related to calcium levels. An abrupt fall in calcium concentration leads to reduction of the production and release of parathormone (PTH) and exacerbates the secondary clinical manifestations, because of hypocalcemia. Phosphorus concentration is inversely related to calcium and is regulated by calcium, PTH and vitamin D.^2^

## OBJECTIVE

The aim of this study was to evaluate the changes in magnesium and phosphorus ion concentrations in patients undergoing thyroidectomy, and to correlate these changes with changes to total calcium.

## METHODS

Patients were evaluated prospectively and were included in the study after signing a free and informed consent statement that was in accordance with the World Health Organization guidelines for research on human beings.^5^

All the patients who underwent thyroidectomy performed by the Head and Neck Surgery Group in the Alpha Institute of Gastroenterology, Hospital das Clínicas, Universidade Federal de Minas Gerais (UFMG), between September 2000 and December 2005, were eligible for inclusion in the study. All the patients included in this study had a formal surgical indication for thyroidectomy, independently of the extent of the surgery.

Patients who chose not to participate in the study, those with an incomplete preoperative assessment, those who did not return for their follow-up appointments and those who presented preoperative hypo or hyper magnesemia/phosphatemia or preoperative hyperparathyroidism (as shown through calcium ion and PTH measurements) were excluded from this study.

In addition to the routine examinations that are performed prior to any thyroidectomy, the preoperative assessment for these patients included measurement of total calcium, phosphorus and magnesium ion levels in the blood. The postoperative assessment included measurement of total calcium, phosphorus and magnesium ion levels in the blood, 24 hours and 48 hours after the operation. All the blood samples were taken at 6:00 a.m.

Patients who presented postoperative clinical manifestations of hypocalcemia, as confirmed through laboratory tests, received oral calcium carbonate. The initial dose was 2.0 grams every six hours, and this was adjusted in accordance with the clinical and laboratory follow-up. In treatment-resistant cases (high doses of oral calcium carbonate with no clinical improvement), oral vitamin D was also administered. Severely symptomatic patients received intravenous calcium gluconate, as well as oral calcium carbonate, until the clinical manifestations had disappeared.

Patients were evaluated according to the presence or absence of postoperative hypocalcemia. This variable was correlated with preoperative phosphorus (P) and magnesium (Mg) measurements and serum concentrations of postoperative magnesium and phosphorus ions, in samples taken on the first and second day after thyroidectomy;

Concentrations that were below the reference values for the ions evaluated were considered to be cases of hypocalcemia, hypophosphatemia and hypomagnesemia. The laboratory tests were performed at the Clinical Pathology Laboratory of Hospital das Clínicas, UFMG, and included the following:

- Total calcium – reference value: 8.4 mg/dl to 10.2 mg/dl.
- Phosphorus – reference value: 2.5 mg/dl to 4.5 mg/dl.
- Magnesium – reference value: 1.6 mg/dl to 2.3 mg/dl.

The data were analyzed using the Statistical Package for the Social Sciences (SPSS), version 13.0. The significance level was taken to be 5%.

## RESULTS

The study included 359 patients, of both genders and all age groups, who underwent thyroidectomy performed by the Head and Neck Surgery Group in the Alpha Institute of Gastroenterology of the University Hospital of UFMG, between September 2000 and December 2005.

The complete outpatient follow-up in accordance with the proposed method was performed in the cases of 333 patients. The other 26 patients were excluded.

Among these 333 patients, 29 (8.7%) were male and 304 were female (91.3%). Their age ranged from 8 to 88 years, with a mean of 45 ± 15 years and a median of 46 years.

The mean values for total calcium, phosphorus and magnesium before the operation and on the first and second postoperative days are shown in Table 1.

**Table 1.** Mean total calcium, phosphorus and magnesium levels before the operation and on the first and second postoperative days (POD)

	Total calcium (mg/dl)	Phosphorus (mg/dl)	Magnesium (mg/dl)
Before operation	9.23 ± 0.54	3.48 ± 0.58	1.96 ± 0.26
First POD	8.36 ± 0.40	3.36 ± 0.72	1.83 ± 0.42
Second POD	8.31 ± 0.78	3.36 ± 0.79	1.89 ± 0.40

Reference values: total calcium, 8.4 to 10.2 mg/dl; phosphorus, 2.5 to 4.5 mg/dl; magnesium, 1.6 mg/dl to 2.3 mg/dl.

**Figure 1.**
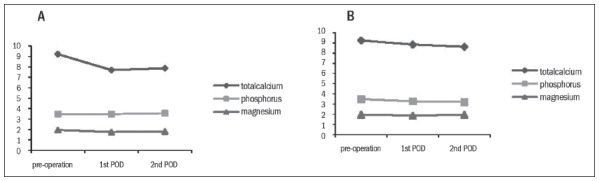
Mean total calcium, phosphorus and magnesium levels before the operation and on the first and second postoperative days (POD), among patients with hypocalcemia (A) and without hypocalcemia (B) after the surgery.

Among the hypocalcemic patients, the mean values for total calcium, phosphorus and magnesium on the first postoperative day were, respectively, 7.69 ± *0.74 mg/dl, 3.47* ± *0.77 mg/dl and 1.75* ± *0.46 mg/dl. On the second postoperative day, the values were 7.87* ± *0.85 mg/dl, 3.56* ± *0.93 mg/dl and 1.81* ± 0.46 mg/dl (Figure 1A).

Among the patients without hypocalcemia, the mean values on the first postoperative day were, respectively, 8.83 ± *0.52 mg/dl, 3.27* ± *0.68 mg/dl and 1.88* ± *0.39 mg/dl. On the second postoperative day, the values were 8.62* ± *0.55 mg/dl, 3.21* ± *0.65 mg/dl and 1.95* ± 0.34 mg/dl (Figure 1B).

Among the patients without postoperative hypocalcemia (n = 197), the total calcium levels were lower on the first and second postoperative days than before the operation, but the difference was only statistically significant on the second day (P = 0.000). Among the patients with postoperative hypocalcemia (n = 136), the drop in the levels was significant on both days following surgery (P = 0.000) (Table 2).

**Table 2.** Comparison of mean total serum calcium levels (mg/dl) before the operation (pre) and on the first and second postoperative days (POD), among 333 patients, with or without hypocalcemia

Group	Variables	Difference between means (mg/dl)	P
Without hypocalcemia	TotCa pre vs. TotCa 1^st^ POD	0.41	0.265
	TotCa pre vs. TotCa 2^nd^ POD	0.62	0.000
With hypocalcemia	TotCa pre vs. TotCa 1^st^ POD	1.51	0.000
	TotCa pre vs. TotCa 2^nd^ POD	1.34	0.000

TotCa = total calcium; vs. = versus.Statistical method = Student’s t test.

There was a statistically significant drop in blood phosphorus levels on these two days following thyroidectomy, compared with preoperative levels, among the patients without hypocalcemia (P = 0.000). Among the patients who developed hypocalcemia, there was a significant drop in plasma phosphorus on the first postoperative day (P = 0.006) and an increase (which was also significant) on the second day (P = 0.012), in relation to preoperative phosphorus levels (Table 3).

**Table 3.** Comparison between mean serum phosphorus levels (mg/dl) before the operation (pre) and on the first and second postoperative days (POD), among 333 patients, with or without hypocalcemia

Group	Variables	Difference between means (mg/dl)	P
Without hypocalcemia	P pre vs. P 1^st^ POD	0.18	0.000
	P pre vs. P 2^nd^ POD	0.26	0.000
With hypocalcemia	P pre vs. P 1^st^ POD	0.05	0.006
	P pre vs. P 2^nd^ POD	-0.04	0.012

P = phosphorus; vs. = versus. Statistical method = Student’s t test.

A significant drop in postoperative magnesium levels was also observed on both the first and second postoperative days following thyroidectomy, among the patients who developed hypocalcemia (P = 0.000). Among the patients without hypocalcemia, the drop in magnesium levels was significant on the first day (P = 0.017), but no significant difference was noted on the second day (P = 0.876) (Table 4).

**Table 4.** Comparison between mean serum magnesium levels (mg/dl) before the operation (pre) and on the first and second postoperative days (POD), among 333 patients, with or without hypocalcemia

Group	Variables	Difference between means (mg/dl)	P
Without hypocalcemia	Mg pre vs. Mg 1^st^ POD	0.43	0.017
	Mg pre vs. Mg 2^nd^ POD	0.01	0.876
With hypocalcemia	Mg pre vs. Mg 1^st^ POD	0.22	0.000
	Mg pre vs. Mg 2^nd^ POD	0.15	0.000

Mg = magnesium; vs. = versus.Statistical method = Student’s t test.

## DISCUSSION

Phosphorus and magnesium ions have a direct relationship with calcium metabolism. Plasma calcium concentrations frequently present disorders following thyroidectomy.

The adult human body contains approximately 600 g of phosphorus (1.0% of total body weight), of which 85.0% is in the skeleton, while the remaining 15.0% can be found in extracellular fluids, in the form of inorganic phosphate, and in soft tissue, in the form of phosphate esters.^1^

Intestinal and renal absorption and excretion of phosphorus are related to PTH concentration. PTH increases vitamin D concentration, which is responsible for stimulating active absorption of this phosphorus in the intestines. In the kidneys, PTH acts directly on proximal tubules, thereby reducing phosphorus reabsorption and increasing phosphaturia.^6^

The drop in serum concentration of phosphorus in response to PTH stimulation occurs more promptly than does the increase in calcium. Therefore, monitoring of phosphorus in hypocalcemic patients who require calcium supplementation may provide an early indication of regulation of parathyroid gland function.^7^

Magnesium deficiency reduces the PTH effect in the kidneys and bones and increases its degradation in the liver and kidneys. Therefore, hypocalcemic patients with magnesium deficiency will present relative hypoparathyroidism.^8^

Magnesium is also involved in the metabolism and activity of vitamin D. Patients with hypocalcemia and hypomagnesemia are resistant to large doses of vitamin D, because of reductions in PTH secretion and renal resistance to this hormone. Furthermore, oral administration of vitamin D does not increase blood calcium levels.^9^

Hypomagnesemia and hypocalcemia occurring together increase the symptoms in patients. Plasma calcium correction without concurrent normalization of magnesium may prolong the clinical manifestations.^7,10^

Temporary hypoparathyroidism leads to a reduction in renal reabsorption of magnesium, and expansion of the extracellular volume increases magnesium excretion.^10^ According to Wilson et al.,^10^ 10.0% of patients who undergo total thyroidectomy develop hypomagnesemia and hypocalcemia.

Our results showed that the mean total calcium level in patients who underwent thyroidectomy decreased. This confirms the results published by other authors, thus underscoring the influence of hemodilution on total calcium concentration during and following surgery. Measurements on ion concentrations on the first two days following the operation showed recuperation, albeit partial at times, of fluid volume and plasma ion concentrations. Aside from hemodilution, significant and prolonged reduction of calcemia may be caused by temporary disorders of the parathyroid glands, and consequently, by hypoparathyroidism. Although routine measurement of PTH following thyroidectomy is not available at our service, hypoparathyroidism can be suspected when there is a drop in calcium levels, especially when patients require oral supplementation.

There was a significant drop in plasma phosphorus levels in patients without hypocalcemia, on the first two days following surgery in relation to preoperative levels, which suggests that hemodilution may have an important role. Among the patients with hypocalcemia, a significant drop in phosphorus levels was observed on the first postoperative day. However, possibly because of lack of PTH stimulation caused by parathyroid gland disorders, there was a reduction in renal excretion of phosphorus and a progressive increase in blood levels of this ion, in contrast to the reduction in calcium. Therefore, plasma phosphorus levels rose to significantly higher levels on the second postoperative day.

The patients who presented hypocalcemia presented significantly lower magnesium levels on the first two days following the operation, in comparison with preoperative levels. Patients without hypocalcemia had a significant drop in magnesium levels on the first postoperative day, with a return to baseline levels on the second postoperative day, which might indicate volume reestablishment. However, both in the patients with and in those without hypocalcemia, the serum magnesium levels remained within the reference values, thus making it difficult to evaluate their role in postoperative hypocalcemia. Even in patients with symptoms of hypocalcemia and significant reductions in calcium levels, the blood magnesium levels remained within their reference values and probably did not influence the reduction in calcium levels and hypocalcemia symptoms.

## CONCLUSIONS

In conclusion, despite the postoperative changes, neither the magnesium nor the phosphorus levels had any role in post-thyroidectomy calcemia. The magnesium concentration varied according to total calcium levels, while the phosphorus concentration was inversely proportional.
